# Chronic kidney disease: detect, diagnose, disclose—a UK primary care perspective of barriers and enablers to effective kidney care

**DOI:** 10.1186/s12916-024-03555-0

**Published:** 2024-08-15

**Authors:** Stuart Stewart, Philip A. Kalra, Tom Blakeman, Evangelos Kontopantelis, Howard Cranmer-Gordon, Smeeta Sinha

**Affiliations:** 1 The University of Manchester, Centre for Primary Care & Health Services Research, Manchester, UK; 2 Donal O’Donoghue Renal Research Centre, Northern Care Alliance NHS Foundation Trust, Manchester, UK; 3 Rochdale Care Organisation, Northern Care Alliance NHS Foundation Trust, Manchester, UK; 4 Manchester Academic Health Science Centre, The University of Manchester, Manchester, UK

**Keywords:** Chronic kidney disease, Primary care, Detection, Screening, Population health, COVID-19, Incentivised care, Primary care networks, Integrated care systems

## Abstract

Chronic kidney disease (CKD) is a global public health problem with major human and economic consequences. Despite advances in clinical guidelines, classification systems and evidence-based treatments, CKD remains underdiagnosed and undertreated and is predicted to be the fifth leading cause of death globally by 2040. This review aims to identify barriers and enablers to the effective detection, diagnosis, disclosure and management of CKD since the introduction of the Kidney Disease Outcomes Quality Initiative (KDOQI) classification in 2002, advocating for a renewed approach in response to updated Kidney Disease: Improving Global Outcomes (KDIGO) 2024 clinical guidelines. The last two decades of improvements in CKD care in the UK are underpinned by international adoption of the KDIGO classification system, mixed adoption of evidence-based treatments and research informed clinical guidelines and policy. Interpretation of evidence within clinical and academic communities has stimulated significant debate of how best to implement such evidence which has frequently fuelled and frustratingly forestalled progress in CKD care. Key enablers of effective CKD care include clinical classification systems (KDIGO), evidence-based treatments, electronic health record tools, financially incentivised care, medical education and policy changes. Barriers to effective CKD care are extensive; key barriers include clinician concerns regarding overdiagnosis, a lack of financially incentivised care in primary care, complex clinical guidelines, managing CKD in the context of multimorbidity, bureaucratic burden in primary care, underutilisation of sodium-glucose co-transporter-2 inhibitor (SGLT2i) medications, insufficient medical education in CKD, and most recently – a sustained disruption to routine CKD care during and after the COVID-19 pandemic. Future CKD care in UK primary care must be informed by lessons of the last two decades. Making step change, over incremental improvements in CKD care at scale requires a renewed approach that addresses key barriers to detection, diagnosis, disclosure and management across traditional boundaries of healthcare, social care, and public health. Improved coding accuracy in primary care, increased use of SGLT2i medications, and risk-based care offer promising, cost-effective avenues to improve patient and population-level kidney health. Financial incentives generally improve achievement of care quality indicators – a review of financial and non-financial incentives in CKD care is urgently needed.

## Background

Chronic kidney disease (CKD), characterised by an often progressive and irreversible loss of kidney function, is a highly prevalent global and national public health problem [1]. CKD is classified according to its cause and into stages according to a patient’s estimated glomerular filtration rate (eGFR) and albuminuria [2]. Globally, 700 million people (9.1% prevalence) are estimated to have any stage of CKD [3]. In England, adult CKD (Stages G3-G5) prevalence in primary care was 4.5% as of June 2023, according to CVDPREVENT [4] – a national primary care electronic health record (EHR) auditing tool with coverage of 95% of GP practices. However, prevalence estimates vary depending on data source, time period and inclusion criteria (Appendix). Whilst several major public health problems have seen dramatic reductions in global mortality rates since 1990, CKD has not, and is consequently predicted to be the fifth leading cause of death globally by 2040 [3]. Earlier stages of CKD (G1-G2) are usually asymptomatic, however, as kidney function declines the risk of acute kidney injury (AKI), cardiovascular disease and death rises [2]. Between 2009–2010 in England alone, CKD was associated with 7,000 extra strokes, 12,000 extra myocardial infarctions, and 45,000 premature deaths [5]. Although only 1–2% of patients with CKD will ever progress to kidney failure [2] (KF– G5 with eGFR < 15 ml/min), the cost of managing KF with dialysis and kidney transplants in the National Health Service (NHS) was estimated at £1.34 billion in 2023 [6]; dialysis alone is estimated to cost the NHS £34 k per patient per annum – more than triple the annual value of a state pension [6]. Patients with CKD also face significant health inequities and health inequalities. For example, Black and ethnic populations are up to five times more likely to progress to KF requiring kidney replacement therapy (KRT) yet wait longer for kidney transplants [7, 8]. Female patients are more likely to be diagnosed with CKD yet male patients are at greater risk of progression to KF [7, 8]. Younger patients with CKD are less likely to receive kidney protective and cardiovascular risk-reducing medications (e.g. renin-angiotensin system inhibitors (RASi), and statins), yet older patients are less likely to have their CKD diagnosis recorded [7, 9], and are less likely to receive a kidney transplant which in turn results in a longer duration of dialysis compounding their already increased cardiovascular risk [7, 8]. Patients of lower socioeconomic status and patients [10] with significant mental health disease are at increased risk of CKD and progression to later stages of CKD [7]. Patients of lower socioeconomic deprivation are also more likely to be diagnosed late reducing opportunities for risk reduction [7]. A public health problem of this magnitude clearly requires a robust and effective detection and monitoring strategy.

The aim of this review is to identify both key barriers and enablers to effective CKD care since the introduction of the Kidney Disease Outcomes Quality Initiative (KDOQI) classification of CKD in 2002. In doing so, we argue for a renewed approach to managing CKD at both the patient and population level in response to these barriers and enablers, to a changing healthcare landscape, and updated Kidney Disease: Improving Global Outcomes (KDIGO) 2024 guidelines [2] for managing CKD.

To identify relevant sources and studies for this review, we conducted a thorough literature search using PubMed, Google Scholar, CINAHL, and grey literature sources. We used a combination of Medical Subject Headings (MeSH) terms and free text keywords that linked to our main aim and selected these based on a review of high impact papers with subsequent refinement through iterative searches. The search was limited to studies published in English or with an English abstract between January 2000 and May 2024. The search terms included “chronic kidney disease”, “primary care”, “general practice”, “family medicine”, “detection strategies”, “diagnosis”, “disclosure”, “non-disclosure”, “management”, “screening”, “primary care networks”, “integrated care systems”, “incentives”, “financial incentives”, “quality and outcomes framework”, “health inequalities”, “barriers”, and “enablers”. We reviewed the full text of relevant articles and their reference lists to identify further relevant material.

## Detection

The introduction of the KDOQI classification for CKD in 2002 marked a significant advance in the management of CKD, providing clinicians with a structured approach to identifying and categorising kidney disease [11]. The classification system was later adapted by KDIGO for a global audience [11] and incorporated into the first guidelines by the National Institute for Health and Care Excellence (NICE) in 2006 for CKD management. At the same time, detection, diagnosis, and management was also financially incentivised in UK primary care via the Quality and Outcomes Framework (QOF) from 2006 to 2015 [5, 12].

Generally, the medical community embraced the KDIGO classification system – facilitating a standardised approach to the evaluation and stratification of kidney disease [1]. Clinically, the system helped link disease stages to specific actions including prescribing, sick day rules and monitoring, aiding discussions around prognosis and shared decision-making. However, with widespread application, concerns regarding overdiagnosis and overmedicalisation of natural ageing as a newly defined ‘disease’ prompted debate on the utility of the classification system [13]. Critics pointed out the disproportionate prevalence of early-stage CKD compared to KF, inaccuracies in glomerular filtration rate (GFR) estimation methods like the Modification of Diet in Renal Disease (MDRD) equation, and the characterisation of early CKD stages as risk factors or pre-disease states, akin to pre-diabetes [1]. Further issues included the system's lack of coherence—where some patients in early stages faced higher health risks than those in later stages, and the thresholds not reflecting GFR and albuminuria variations across different ages, sexes, and ethnicities [1]. These issues underscored the need for a more nuanced understanding of CKD, recognising the diversity in patient risk profiles and the variability in kidney function across different populations.

In 2012, the accuracy of GFR estimation improved significantly with the introduction of the Chronic Kidney Disease Epidemiology Collaboration (CKD-EPI) equation [14] when compared to the MDRD equation. Bolstered by population-level studies [15], use of the CKD-EPI equation caused a notable decline in the classification of individuals under 70 years old into CKD stages G3-G5—a 7.5% relative decrease, dropping from 15.7% to 14.5% [15]. Conversely, there was a slight rise in the prevalence of CKD stages G3-G5 among men over 70, from 33.3% to 35.5%. Crucially, this research suggested that the CKD-EPI equation could potentially lower the overall CKD prevalence by up to 1.9 million cases and reduce the prevalence of CKD stages G3-G5 by an additional 200,000 cases [15]. Nevertheless, and illustrating the complexity of detecting and defining kidney disease at a population level, recent research examining the application of the 2021 CKD-EPI equation (without race modifier for Black people) in a population of 1.2 m predominantly White individuals in Stockholm, curiously resulted in 36.2% of patients with CKD G3a-5 being reclassified into a higher stage [16]. Therefore, the European Kidney Function Consortium (EKFC) have developed the EKFC equation [17] to address the age and race limitations inherent to CKD-EPI whilst having equal performance for Black and non-Black populations [18].

Despite these advances in addressing overdiagnosis, the complexity of CKD guidelines and their translation into financial incentives via QOF raised broader issues related to increasing workloads and administrative pressures on general practitioners (GPs) [19]. Within this framework, aspects of CKD care began to be viewed as mere bureaucratic formalities [12], even though other studies indicated that CKD was still underdiagnosed [20]. By 2015, incentivised CKD care through QOF was discontinued without any national strategy to assess the impact of this decision on quality of care (QoC) or to ensure the maintenance of existing care standards. This raises the question: how did removal of CKD care from QOF impact testing and detection?

**Fig. 1:**
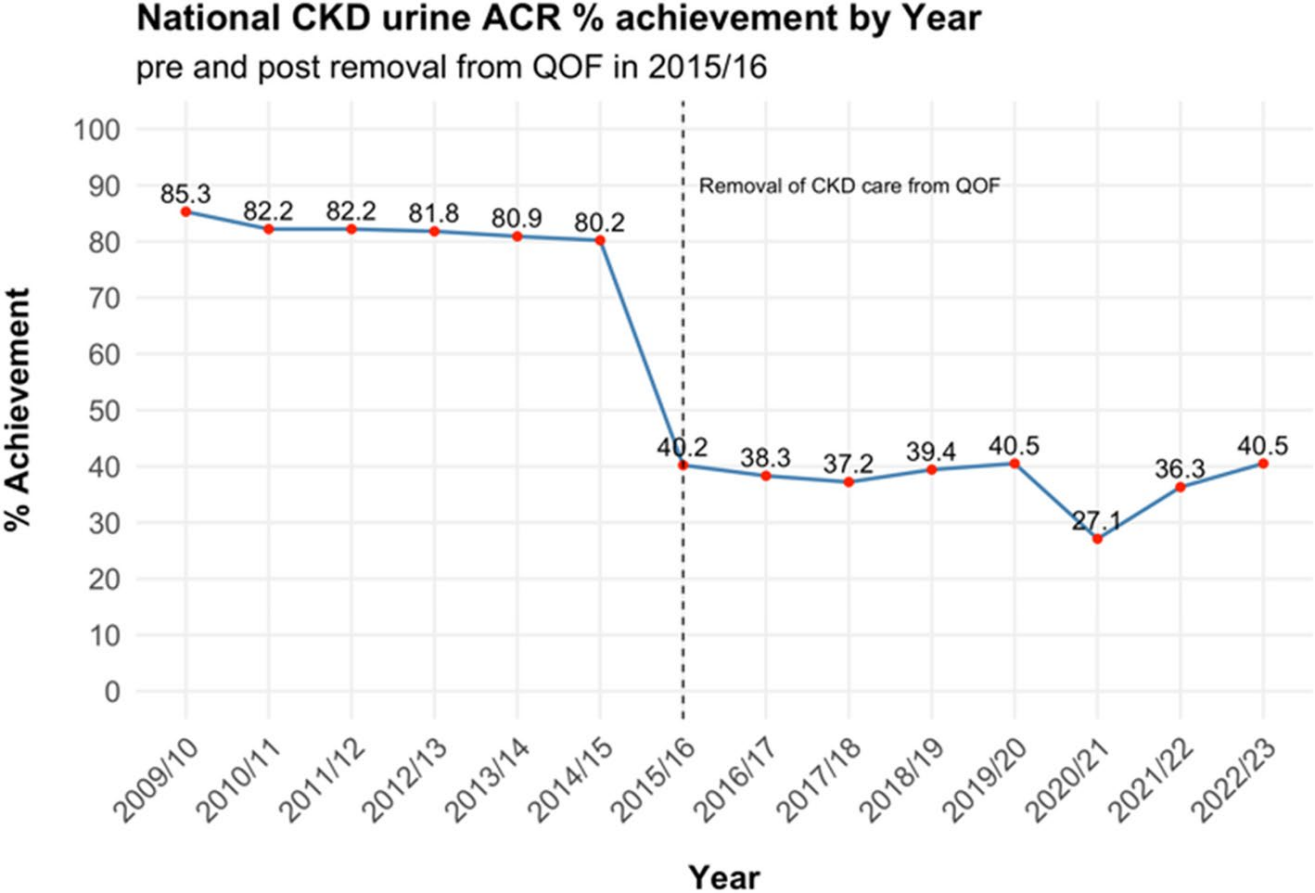
National CKD urine ACR % achievement by year. Created from NHS Digital QOF, Indicators No Longer in QOF (INLIQ) [21–28], and CVDPREVENT [29] source data. Note: Indicator CKD004 was known as CKD006 from 2009/10 to 2012/13

Albuminuria testing (QOF indicator CKD004 / CVDPREVENT indicator CVDPCKD004) – critical for the diagnosis, classification and prognosis of CKD – fell dramatically from ~ 80% to ~ 40% following withdrawal from QOF in 2015/16 (Fig. 1). Data from the national CKD audit (NCKDA) in 2017 raised even more concern highlighting proteinuria was measured in less than 15% of non-diabetic patients and only 54% of patients with diabetes [9] – challenging the assumption that effective CKD care could be absorbed into other financially incentivised chronic disease care in primary care. Whilst these findings directly pertain to patients *with CKD*, it is reasonable to expect a similar if not worse picture of proteinuria testing in patients *at risk of CKD.* Without measuring proteinuria, our ability to accurately identify those at greatest risk, especially patients with early stages of CKD (G1-G2) is significantly undermined.

Research by Wilding et al., measuring changes in several CKD and non-CKD QoC measures following withdrawal from QOF adds more colour to this picture [30]. Specifically, blood pressure control to the target of < 140/85 mmHg, which had been on an upward trend from 58.24% in 2007/08 to 68.92% in 2014/15, experienced a decline of 7.78% in 2015/16 to 61.14% (Table 1). This downturn was not just a matter of fewer CKD patients reaching the target blood pressure post-QOF withdrawal but also reflected an increase in the number of patients who did not have their blood pressure measured at all (noted as CKD002 missed in Table 1). Such trends are particularly alarming in light of the concurrent decrease in uACR testing, increasing the potential for accelerated progression of kidney disease and missed opportunities for early intervention. Morales et al. [31] highlighted through a controlled interrupted time series analysis a worsening of performance in 12 of 16 QoC indicators by one year and 10 of 16 QoC indicators by three years across several chronic diseases after withdrawal of QOF in Scottish primary care, compared with England [31]. Whilst the authors did not measure the impact on CKD QoC indicators, withdrawal did impact complex care processes (diabetes, mental health disease) and therefore CKD care (also a complex care process) may well have been adversely impacted post-QOF [31]. When considering these findings through the lens of behavioural psychology, work from Lepper et al. [32] in 1973, highlights removal of extrinsic rewards undermines internal motivation and can result in not only loss of positive behaviours (detecting and diagnosing CKD) but also worsening of performance in general (managing pre-existing CKD).

**Table 1:** Quality and outcomes framework CKD indicator (CKD002 and CKD004) performance over time in primary care in England. Date sources – Wilding et al. [30], NHS Digital QOF and INLIQ data [21–25, 28], and CVDPREVENT [29] data

Year	QOF indicators			
	CKD002 achieved	CKD002 not achieved	CKD002 missed	CKD004^a^ achieved
2007/08	58.24%	31.22%	8.68%	-
2008/09	59.73%	29.83%	8.12%	-
2009/10	60.68%	29.66%	7.15%	85.3%
2010/11	62.43%	28.05%	6.79%	82.2%
2011/12	63.40%	26.76%	6.96%	82.2%
2012/13	63.60%	25.36%	7.83%	81.8%
2013/14	70.72%	19.45%	7.27%	80.9%
2014/15	68.92%	19.86%	8.37%	80.2%
2015/16	61.14%	24.18%	10.72%	40.2%
2016/17	61.87%	25.26%	10.11%	38.3%

Key: CKD002: The percentage of patients on the CKD register in whom the last blood pressure reading (in the preceding 15 months) is 140/85 mm/Hg or less; CKD004: The percentage of patients on the CKD register whose notes have a record of a urine ACR or PCR test in the preceding 15 months; ^a^CKD004 was CKD006 from 2009/10 to 2012/13

Policymakers and leaders must therefore consider, prior to implementation, how population-level changes to care (such as withdrawal of financially incentivised care) could impact patient and health system outcomes including whether health inequalities will be created or widened. In the interests of transparency and due diligence, these analyses should be published. Decision analysis – popular in business, economics and public health, used to forecast the consequences of different COVID-19 lockdown exit strategies in the UK [33], could help policymakers and leaders forecast unforeseen consequences of withdrawal of financial incentives prior to implementation.

The predictive power of proteinuria in kidney disease progression risk is well-established and beyond dispute [2]. The question is not whether to measure and monitor proteinuria but how to effectively implement its measurement. The integration of such tests within financially driven incentives via QOF within primary care proved to be unacceptable. Thus, it raises the question of whether a population health strategy, leveraging the organisational structures of Primary Care Networks (PCNs) – introduced in 2019 in England and tasked with providing integrated community-based clinical and social care, might offer a more efficacious approach. Addressing major public health challenges necessitates population-level strategies, which are foundational to the operations of Integrated Care Systems (ICSs) and PCNs; these networks have already demonstrated their potential in effectively managing major public health problems, as seen in their critical role in the national COVID-19 vaccination program [34].

Considering the role of screening in the detection of kidney disease—the NHS Screening Committee does not currently endorse population screening, citing concerns over cost-effectiveness and clinical efficacy [35]. This position stems in part from the absence of treatments proven to significantly slow the progression of CKD [35]. However, it’s crucial to acknowledge the aetiological complexity of CKD as a condition. Unlike more homogeneous diseases like aortic aneurysms (where screening is endorsed), CKD encompasses a wide variety of underlying causes that contribute to kidney function decline, making it difficult to neatly apply NHS screening criteria. Notably, longitudinal research in UK primary care from 2013 to 2017 revealed 44% of adults aged 60 + had undiagnosed CKD, even after excluding terminally ill and older adults unable to consent [36]. Such data underscores the high yield and potential benefits of targeted screening in high-risk groups – where CKD screening is recommended [2]. Given the prevalence of CKD and its human and economic consequences [5, 6], identifying and screening those at greatest risk is imperative. The emergence of new treatments, such as sodium-glucose co-transporter 2 inhibitors (SGLT2i), which not only aid in glycaemic control but also significantly reduce risk of cardiovascular events, death, and kidney disease progression risk [37], should therefore prompt a re-evaluation of the current stance on CKD screening [38].

## Diagnosis

Central to the debate around overdiagnosis is the question – what is the utility of a diagnosis of CKD? Diagnosis encompasses not only the identification of CKD but also the subsequent recording and coding (application of a diagnostic clinical code; e.g., SNOMED CT, ICD-10, retired Read codes) [39] of the diagnosis within primary care EHR systems *and* disclosure of the diagnosis with patients. Here we consider diagnosis and coding before disclosure. Coding CKD in the EHR is associated with numerous benefits for both clinicians and patients. In fact, coding a diagnosis of CKD is one of the single most cost-effective tools at a clinician’s disposal owing to the myriad of protective downstream effects of applying diagnostic clinical codes [9]—automated patient recall for routine CKD reviews; automated recommendations to perform kidney function testing in line with guidelines; cross-disease EHR alerts to encourage kidney function testing in at-risk groups; automated and timely prescribing warnings to promote kidney protective medications in line with clinical observations (e.g., blood pressure) and discourage harmful medication prescribing (NSAIDs [40]); as well as vaccination eligibility alerts. Interestingly, not only does neglecting to code a diagnosis not afford these positive benefits, it is also associated with major adverse health outcomes for patients, revealed in the NCKDA; patients with uncoded CKD stages G3-G5 experience up to 500% higher mortality risk, compared to patients whose CKD is appropriately coded, when matching for age, sex, disease stage and comorbidities [41]. Furthermore, patients with uncoded CKD are also at greater risk (increasing risk as eGFR decreases) of unplanned hospital admissions and AKI [41]. Although associations between uncoded CKD and the presence of other significant health conditions, such as dementia and advanced cancer [9] may theoretically amplify this risk, the magnitude of risk increase is nevertheless substantial. With this in mind, the NCKDA findings highlights a concerning issue: not only are there clear inconsistencies across England and Wales in how CKD is identified in primary care but there is a wide disparity in how a CKD diagnosis is recorded (coded) for patients with biochemical evidence of CKD (reduced eGFR and raised uACR), with rates varying from 0 to 80% [9].

Considered from another angle, the importance of being able to effectively identify CKD at both the individual and population level came into sharp focus as CKD emerged as a major risk factor for death from acute COVID-19 infection in adults [42, 43]. Moreover, patients with CKD require ‘special attention’ owing to increased mortality risk [43] (Table 2), tailored shielding advice [44, 45], and reduced vaccine efficacy [46]. Here, the power of a diagnostic clinical code emerges – used as a key instrument by NHS England and GP Practices (as well as specialist nephrology services) for identifying patients with CKD for shielding advice [45], care planning [47, 48], and vaccination priority [49]. How then, were patients with uncoded CKD (Table 2: 30.1% of CKD G3-G5 [50]) expected to receive such lifesaving care and what impact did this failure to code a diagnosis of CKD have on patient outcomes? Although unknown, given uncoded CKD is associated with increased all-cause mortality risk [41], it is reasonable to assume uncoded CKD may also be associated with increased COVID-19 mortality risk. With such compelling and clinically plausible associations between uncoded CKD and adverse health outcomes [41], why do so many patients with biochemical evidence of CKD [9] not have a diagnostic clinical code? Research shows an extensive list of barriers to effective CKD care which influence diagnosis and coding (summarised in Table 3). Clinician concern regarding overmedicalisation is an often-cited barrier to making a diagnosis, however, a less apparent but vitally important barrier (and in itself an enabler) to address is clinicians’ lack of awareness of the associated benefits of coding a diagnosis and the harm associated with not. In fact, coding a diagnosis of CKD is an equity imperative, especially in the context of future pandemic preparedness.

**Table 2:** Mapping of CKD stages G3-G5 from NCKDA data [50] to hazard ratios regarding risk of death from COVID-19 from OpenSAFELY data [42]

	**National CKD Audit data** [50]** from 8.24 m adults in primary care in England and Wales**			**OpenSAFELY data** [42]** from 17 m adults in primary care in England**		
	*n* with biochemical CKD at round 1 of NCKDA	% coded for CKD	% uncoded for CKD	**Fully adjusted hazard ratios (95% CIs)**		
				Primary analysis	Early censoring at 6/4/2020	Adjusted for ethnicity using multiple imputation
**CKD Stage 3a**	160,100	60.8	39.2	1.33 (1.28–1.40)	1.49 (1.36–1.63)	1.33 (1.27–1.39)
**CKD Stage 3b**	78,855	82.5	17.5			
**CKD Stage 4**	17,224	89.7	10.3	2.52 (2.33–2.72)	2.98 (2.57–3.43)	2.50 (2.31–2.70)
**CKD Stage 5**	3,254	90.3	9.7			
**All CKD 3–5**	259,433	69.9	30.1

**Table 3:** Summary of primary care practitioner’s (PCP’s) reported barriers to effective CKD care in primary care considered as a potential barrier to detection, diagnosis, disclosure and management [52–58]

		**Potential barrier to**^**a**^			
	**Specific barrier**	**Detection**	**Diagnosis**	**Disclosure**	**Management**
**System Factors**	Limited visit time to care for complex patients	✓	✓	✓	✓
	Lack of standardised quality of care metrics	✓	✓	✓	✓
	Lack of comprehensive clinical information (EHR) systems	✓	✓		✓
	Lack of integration of clinical decision support tools in the EHR	✓	✓	✓	✓
	Long waiting lists for specialist nephrology services		✓		✓
	Poor reimbursement/funding for delivering optimal CKD care	✓	✓		✓
**Primary Care Factors**	PCP’s limited recognition or knowledge of CKD	✓	✓	✓	✓
	PCP’s lack of awareness of CKD guidelines or useful protocols for CKD care	✓	✓	✓	✓
	PCP’s difficulty in assimilating/implementing complex CKD guidelines	✓	✓	✓	✓
	PCP’s experience that CKD risk factors are difficult to manage	✓	✓		✓
	PCP’s beliefs that they are unable to improve CKD	✓	✓	✓	✓
	PCP’s perceptions that proteinuria testing has no impact on management	✓	✓	✓	✓
	Difficulty in integrating proteinuria testing into primary care workflows	✓	✓	✓	✓
	PCP’s concerns regarding overdiagnosis	✓	✓	✓	✓
	PCPs not wanting to worry patients about a diagnosis	✓	✓	✓	✓
	Managing patient and family expectations: *Uncertainty of prognosis; Fostering acceptance of kidney disease severity risk*	✓	✓	✓	✓
	Complexity of conservative medical management: *PCP’s ability to provide best-practice renal care in primary care; integrating multidisciplinary healthcare professionals*				✓
	Negotiating roles and responsibilities when jointly managing patients with specialists				✓
**Patient Factors**	Patients’ limited understanding of CKD and its implications	✓	✓	✓	✓
	Patients unable to afford recommended CKD care				✓
	Patients intolerant of CKD medication side effects				✓
	Insufficient clinical support tools/resources to support self-management				✓

^a^our assimilation of whether a reported barrier is generally a potential barrier to detection, diagnosis, disclosure and/or management; not nessarily considered in source references

At a population level, clinicians can easily detect patients with uncoded CKD using two main methods: within their own practice using population health search tools built into EHR systems where patients are identified by biochemical findings (eGFR and uACR) and clinical codes, and in England – with the CVDPREVENT tool [29] which quickly quantifies the number of uncoded cases of CKD whilst summarising by health inequality measures, at a practice, PCN, ICS, regional and national level. These diagnostic tools are crucial components of a workflow that is integrated with Quality Improvement initiatives, a strategy proven to significantly enhance diagnostic clinical coding for CKD at the population level [51] which sets the stage for diagnosing and coding uncoded cases of CKD at the patient level.

## Disclosure

The debate around disclosure or more precisely, non-disclosure of CKD with patients is complex and nuanced [59, 60]. Research documents several barriers to clinician disclosure of CKD as a diagnosis (Table 3). However, there is also a wealth of evidence showing these barriers can be addressed. One concern among clinicians is the fear of distressing patients regarding a diagnosis that might not noticeably affect their health [56]. Yet, interventional research that embraced a need for ‘minimally disruptive medicine’ [61], demonstrates tailored communication and support through community-based CKD management can enhance health-related quality of life and maintain blood pressure control, without a detrimental effect on patient anxiety [62]. Such community-based interventions align with the establishment of a wider PCN workforce, blending clinical care with social prescribing and broader health and wellbeing initiatives [63]. With a clear move towards a risk-based approach for CKD detection and management, the use of new predictive tools such as the Kidney Failure Risk Equation (KFRE) – incorporated in to NICE CKD 2021 guidelines [64], enable clinicians to predict a patient’s risk of requiring KRT, which can aid in shared-decision making and potentially lessen both clinician and patient anxiety. Interestingly, patients with and without CKD who *inaccurately* report their multiple health conditions have an increased risk of death, after adjusting for age, sex and race [65]. This suggests that accurately understanding one’s own health status has a protective effect further necessitating disclosure of CKD.

Moreover, patients express a desire to be informed of early stages of CKD to facilitate shared decision-making and self-management [66]. On the other hand, clinicians may hesitate to disclose a diagnosis of CKD to some patients with major comorbidities such as metastatic cancer or cognitive impairment. However, disclosing the diagnosis to patients’ next of kin opens up avenues for holistic supportive care. It is crucial to note, coding a diagnosis in the EHR does not equal disclosure. Yet, doing so makes a patient’s kidney impairment visible for all healthcare practitioners and triggers helpful automated alerts within the EHR [8]. The negative implications of non-disclosure, whilst not immediately apparent, also extend to clinicians – a contributing factor for a GP practice being rated inadequate by Care Quality Commissioners and losing its contract to practise [67].

Guidelines increasingly reflect a shift away from terminology that imply disease and deficit towards preserving health, seen in the NICE AKI quality standards [68], alongside integration of CKD into models of maintaining cardiovascular health [69]. Patient-centred research suggests that framing discussions about CKD in terms of maintaining kidney health can encourage patients to take a more active role in their health and by doing so, patients may be more inclined to adopt lifestyle choices and behaviours that preserve kidney function [70].

## Management

To emphasise the advantages of early detection and diagnosis – it is vital to highlight the availability of evidence-based interventions that are proven to reduce mortality, cardiovascular, and kidney disease progression risks. First, RASi (angiotensin-converting enzyme inhibitors or angiotensin II receptor blockers) reduce progression to kidney failure (KF) in patients with albuminuria [2]. Second, SGLT2i are capable of reducing mortality and cardiovascular event risk, as well as reducing the rate of kidney disease progression [2, 71]. Third, lipid lowering therapy with statins reduces mortality and cardiovascular event risk [2, 72]. Importantly, RASi, SGLT2i, non-steroidal mineralocorticoid receptor antagonists and lipid lowering therapies are underutilised in primary care settings in several countries [71, 73, 74]. Fourth, specific therapies tailored to patient’s individual needs and cause of CKD offers additional benefits, e.g., SGLT-2 inhibitors in patients with diabetes, immunosuppressive therapy in patients with lupus nephritis [75]. Whilst these interventions principally benefit individual patients, certain strategies can enhance CKD care at the population level. For example, a collaboration between primary and secondary care physicians to deliver virtual renal clinics have been shown to reduce waiting times for specialist advice (64 to 6 days), reduce outpatient appointments, and improve clinician confidence in managing CKD [76]. Addressing mortality risk, two key levers are available for primary care clinicians to pull immediately – a strategic focus on increasing the uptake of SGLT2i for eligible patients [2] and coding a diagnosis of CKD in all patients with biochemical evidence of CKD [41].

A risk-based approach is central to identifying those patients in greatest clinical need that would benefit from specialist nephrology input, whilst balancing the concerns of overdiagnosis; the KFRE helps to strike this balance. Whilst the KFRE does not predict life-time risk of KRT, doesn’t consider the competing risk of death, and is currently undergoing external validation to ensure it performs fairly in different ethnicities [77] it has been validated and calibrated for a broad UK population prior to implementation and can predict with high accuracy those at greatest risk of KRT [55]. Other risk prediction tools such as KDPredict combine risk of kidney failure with the competing risk of death [78] – an important addition to kidney failure risk prediction which can further support shared decision making and referrals to nephrology. As the KFRE is dependent on eGFR and uACR measurements, primary care clinicians must address the generally poor utilisation of uACR to achieve this risk-based approach. Research has shown that some primary care clinicians lack awareness that measuring uACR can positively impact CKD management [53] therefore medical education is an essential component to influence change. This education must extend to the wider PCN workforce including physician associates and nurse practitioners who receive substantially less training than physicians. When used, the KFRE is helpful in shaping shared decision-making, communicating prognosis, and triggering referral to nephrology for patients with 5-year risk of 5% or more [64, 79].

The complexity and breadth of CKD clinical guidelines hinder effective CKD care highlighting the need for practical solutions. Digital clinical decision tools offer a remedy by distilling extensive guidelines into actionable clinical recommendations. For instance, acknowledging that CKD invariably needs to be placed in the context of supporting people living with multiple long-term conditions, the GP evidence tool used in England [80, 81] condenses key intervention evidence into concise high-yield information on intervention benefits and harms. Similarly, in Canada, the CKD Pathway serves as a streamlined flowchart and guide for diagnosing and managing patients with CKD [82]. Another challenge is the isolated approach of guidelines that focus on single diseases, neglecting the fact that CKD is often diagnosed in the context of multiple long-term conditions. The Cardio-Renal-Metabolic (CaReMe) UK partnership exemplifies a unified approach to this problem by integrating cardiovascular, renal and metabolic disease management [69]. An innovative outcome of this collaboration is a revised type 2 diabetes (T2D) guideline which balances a traditional ‘glycaemia-based approach’ with a cardiorenal risk approach, prioritising SGLT2i to reduce cardiovascular and renal event risk [69] – a strategy now reflected in the latest NICE T2D clinical guidelines [83]. While incorporating elements of CKD care within the broader scope of maintaining vascular health through initiatives like CaReMe has advantages, it is crucial to recognise that it should complement, not replace holistic CKD care that is tailored to individual patient needs.

Financial incentives have demonstrated a mixed but generally positive impact in the management of chronic diseases through care quality indicators and a reduction in health inequalities [84, 85]. Conversely, removing financial incentives in primary care has been linked to a loss of such positive impacts [30, 31, 86]. In the UK, the removal of financial incentives for managing patients with CKD is compounded by the asymmetrical and inadequate weighting of primary care funding in areas of high socioeconomic deprivation [87], where CKD is more prevalent [8]. Whilst there is a dearth of evidence comparing financial incentive models in CKD care in primary care to support policy changes and population-level interventions, British Columbia—Canada is trying a new, evidence-informed approach to managing complex longitudinal care, and in doing so, addressing key system-level barriers (Table 3) to CKD care. For example, a new reimbursement model in primary care financially incentivises primary care clinicians for providing longitudinal care to patients with complex care needs which supports clinicians in spending more time with patients in greatest need [88].

## Conclusions

Reflecting on 22 years of evidence since the KDOQI classification of CKD through to the latest KDIGO CKD 2024 guidelines [2] underlines the complex interplay of detection, diagnosis, and disclosure in the management of CKD within primary care and across health systems. The evidence presented illuminates continuous and iterative attempts to navigate tensions between different stakeholders, objectives, outcomes and evidence which have often hindered as well as helped. Yet, these attempts have sought to find *balance*; balance between the utility of classification systems in their ability to structure *and* constrain our efforts in delivering effective CKD care [89]; balance between delivering individualised patient care and care for populations; balance between optimal CKD care and health system sustainability; balance between the unique skillsets of generalists and specialists; and balance between the tenets of primary care, specialist care, social care and public health. Achieving balance is not only essential for improving patient care and outcomes, but ensuring healthcare professionals remain engaged in delivering effective CKD care in ways that are sustainable for professionals and health systems alike.

Looking ahead, a renewed approach to addressing CKD care across traditional boundaries of healthcare, social care and public health is needed. This approach must be informed by the lessons of the last two decades, prioritise interventions that tackle barriers to detection, diagnosis and disclosure, all whilst responding to the evolving landscape of healthcare delivery. Forecasting and measuring the impact of such interventions and strategies will be pivotal in demonstrating clinical and cost-effectiveness. Beyond making incremental improvements to previous suboptimal care, this approach should seek to deliver personalised care on a scale previously unimagined. ICSs and PCNs and the professionals within them hold the potential to achieving such goals. Achieving these goals requires a collective effort from patients, policymakers, clinicians, and researchers to define and usher in a new era of CKD care.

## Data Availability

Data is provided within the manuscript or supplementary information files.

## Appendix

Table 4

**Table 4:** Estimates of CKD prevalence in England and Wales across different sources over time

**Source**	**Time period**	**Sample population**	**Age**	**Male (%)**	**Female (%)**	**All (%)**	**Estimated CKD stage G3-G5 in England** ^a^
NEOERICA [90]	1998–2003	130,226	18 +	5.8	10.6	8.5	3,640,321 [5]
HSE [91]	2003	7,844	16 +	-	-	7.7	-
CPRD [30]	2006–2007	484,152	18 +	2.73	3.78	3.25	-
QOF [5]	2009–2010	-	18 +	-	-	4.3	1,817,871 [5]
HSE [91]	2009–2010	6,053	16 +	-	-	7.0	2,710,575 [5]
QICKD [92]	2011	930,997	0 +	3.48	7.34	5.4	2,817,104 [5]
OxRen [36]	2013–2017	861	60 +	-	-	13.9	-
National CKD Audit [9, 41]	2015–2016	5,200,000	18 +	-	-	5.5–5.8	~ 1,500,000
HSE [91]	2016	3,766	16 +	-	-	7.3	-
CPRD [30]	2016–2017	400,213	18 +	5.19	7.26	6.24	-
QOF / PHE [93]	2018–2019	-	18 +	-	-	4.1	1,935,056
QOF / PHE [93]	2021–2022	-	18 +	-	-	4.0	1,962,990
CVDPREVENT coded [4]	June 2023	49,352,336	18 +	3.38	4.47	3.93	1,937,683
CVDPREVENT uncoded [94]	June 2023	49,352,336	18 +	0.54	0.68	0.61	301,851
CVDPREVENT combined coded and uncoded [4, 94]	June 2023	49,352,336	18 +	3.92	5.16	4.54	2,239,534

Key: where no values are reported, this is due to a lack of reporting in the source data

^a^estimates from source data

## Abbreviations

AKI: Acute kidney injury
CaReMe: Cardio Renal Metabolic
CINAHL: Cumulative index to nursing and allied health literature
CKD: Chronic kidney disease
CKD-EPI: Chronic Kidney Disease Epidemiology Collaboration
CVDPREVENT: Cardiovascular disease prevent
CPRD: Clinical Practice Research Datalink
eGFR: Estimated glomerular filtration ratio
EHR: Electronic health record
KF: Kidney failure
GP: General Practitioner
HIV: Human immunodeficiency virus
ICBs: Integrated care board/s
ICSs: Integrated care system/s
KDIGO: Kidney Disease: Improving Global Outcomes
KDOQI: Kidney Disease Outcomes Quality Initiative
KFRE: Kidney failure risk equation
MDRD: Modification of Diet in Renal Disease
NCA: Northern Care Alliance
NCKDA: National chronic kidney disease audit
NHS: National Health Service
NICE: National Institute of health and Care Excellence
NSAID: Non-steroidal anti-inflammatory drug
PCN: Primary care network/s
QOF: Quality and outcomes framework
QoC: Quality of care
RASi: Renin-angiotensin system inhibitors
KRT: Kidney replacement therapy
T2D: Type 2 diabetes
SGLT2i: Sodium-glucose co-transporter-2 inhibitor
uACR: Urine albumin creatinine ratio
